# Supplementary material for: Identification of Glu-D1 Alleles and Novel Marker–Trait Associations for Flour Quality and Grain Yield Traits under Heat-Stress Environments in Wheat Lines Derived from Diverse Accessions of Aegilops tauschii
Source: Int J Mol Sci. 2022 Oct 10;23(19):12034. doi: 10.3390/ijms231912034 (PMC9569812; doi:10.3390/ijms231912034)
Supplement: Supplementary file 1 [file ijms-23-12034-s001.zip › Supplementary figures.pdf]

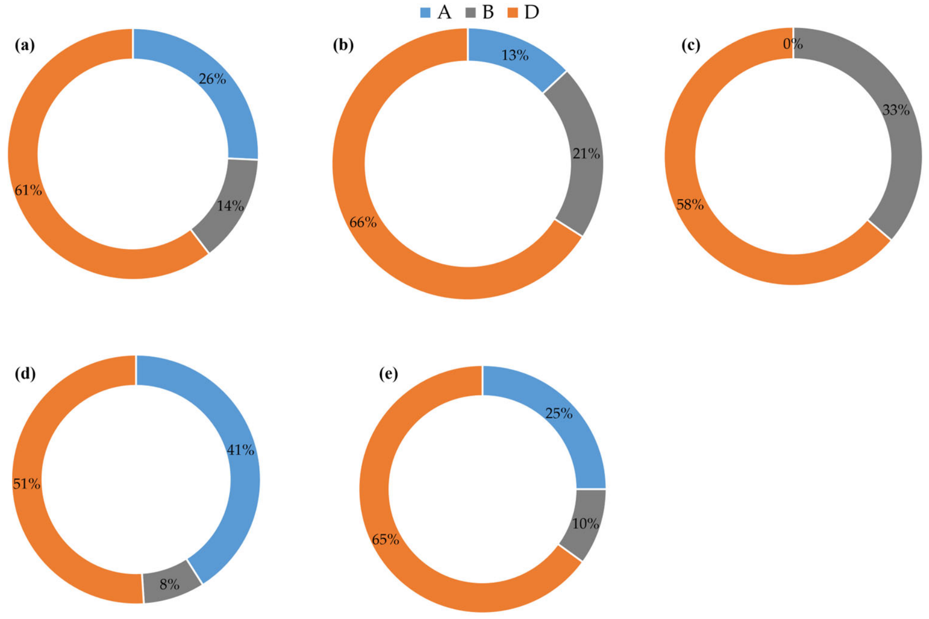

Figure S1: Contribution marker from genome A, B, and D, for all traits across all environments (a), at DON19/20 (b), at HUD19/20 (c), at MED19/20 (d), and at MED18/19 (e).

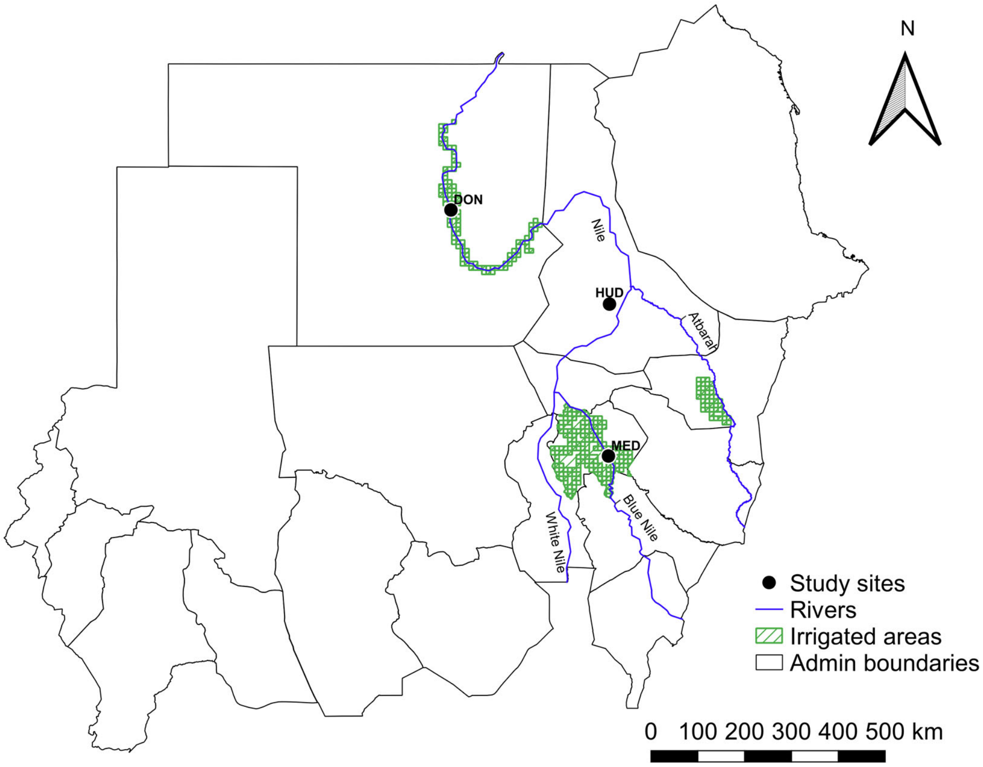

Figure S2. Trials sites in Sudan. DON, Dongola; HUD, Hudeiba; MED, WadMedani.
